# Enhanced corrosion resistance properties of Fe–Al–Cr laser cladding coatings on 1045 carbon steel substrates

**DOI:** 10.1016/j.heliyon.2023.e13855

**Published:** 2023-02-20

**Authors:** Xixi Luo, Jing Cao, Fangli Yu, Yalong Zhang, Jianjiang Tang, Xin Li, Hui Xie

**Affiliations:** a School of Materials Engineering, Xi'an Aeronautical University, Xi'an 710077, China; b State Key Laboratory of Advanced Processing and Recycling of Non-Ferrous Metals, Lanzhou University of Technology, Lanzhou 730050, China

**Keywords:** Fe_3_Al, Cr additive, Coating, Laser cladding, Corrosion resistance

## Abstract

Fe–Al–Cr coatings with different content of Cr additive were prepared on 1045 carbon steel substrates by a laser cladding process. The incorporation of Cr atoms can effectively enhance the corrosion resistance of the coatings. In particular, the Fe–28Al–5Cr laser cladding coating exhibits the best film quality without phase segregation. In addition, the interfacial adhesion between the Fe–28Al–5Cr coating and the 1045 carbon steel substrate is improved. As a result, the Fe–28Al–5Cr laser cladding coating exhibits the best corrosion resistance in a 3.5 wt% NaCl solution under both immersion and electrochemical conditions. However, excessive Cr additive lead to the formation of Al_8_Cr_5_ in the grain boundaries, resulting in inferior corrosion resistance. Therefore, the new findings demonstrated in this work may inspire the design of high-quality coatings with excellent corrosion resistance.

## 1 Introduction

1045 steel has the advantages of relatively high strength, good formability, and low cost, which has been regarded as one of the most important structural materials utilized in various engineering fields [1]. Nevertheless, the corrosion resistance of 1045 steel is poor in moist environment, which shortens its service life and limits its application fields [2]. Since corrosion generally occurs at the surface of a material, the preparation of a protective coating on the surface of 1045 steel is efficient to address this issue [3]. Among all the available coatings, iron aluminide coatings have attracted increasing attention due to their high specific strength, excellent corrosion resistance over a wide range of temperatures, low density, and low cost [4]. In addition to the corrosion resistance of the protective coating itself, the bonding between the protective coating and the substrate is also important. The quality of iron aluminide coatings is dependent on the fabrication process. So far, various methods have been developed to fabricate iron aluminide coatings, such as chemical vapor deposition [5], high-velocity oxy-fuel (HVOF) spraying [6], and pack cementation [7]. However, the fabricated coatings by the above-mentioned methods generally have poor bonding with the substrate, and it is challenging to control the quality of the coatings.

Laser cladding is an emerging surface modification technology that has attracted increasing attention in recent years [[8], [9], [10]]. During the laser cladding process, the feedstock powder is melted by a high energy laser beam, followed by solidification to form a laser cladding coating with a very dense microstructure [11]. The thickness of the laser cladding coating can be easily adjusted. In addition, the laser cladding coating generally has an excellent metallurgical bonding with the substrate [[12], [13], [14]]. It should be mentioned that the performance of the laser cladding coating can be controlled by choosing a wide range of additives in the feedstock powder, which is important to meet different requirements for various applications. For example, a dense and hard Fe–Al coating was successfully deposited on a mild steel substrate by laser cladding [15]. The parameters during laser cladding are important to achieve a high-quality Fe–Al laser cladding coating on mild steel with a specified thickness. Similarly, Al–Cu–Fe coatings were fabricated by a laser cladding process [16]. It was found that the Al–42Cu–5Fe (wt%) composition achieves the best quality of the coating with a hardness of 370 HV_0.2_. Recently, Fe–Cr–Al–Si coatings were prepared using laser cladding technology and their corrosion resistance in a eutectic 75 wt% Na_2_SO_4_ + 25 wt% K_2_SO_4_ salt at 800 °C in air were measured [17].

With the development and utilization of the ocean, the exploration of Fe–Al coatings with excellent corrosion resistance in the mimic marine environment is very important [18]. Some previous works studied the aqueous corrosion behaviors of Fe–Al coatings at room temperature. However, all reported Fe–Al coatings were prepared by other methods such as pack aluminizing method [19], plasma cladding [20], friction stir processing [21], or mechanical alloying [22]. For example, Fe–Al coatings were fabricated by pack aluminizing on low-carbon steel at different temperatures [19], which exhibits better corrosion resistance than the Q235 steel substrate due to the formation of passive ﬁlms. Pitting formation can be observed on the corrosive surface, and the Fe–Al coating with higher content of Fe_2_Al_5_ has better corrosion resistance. Fe–Al coatings were prepared via a plasma cladding method using nitrogen as protective and reactant gas on Q235 substrates [20]. A compact and stable passive film was formed on the surface of the coating, which can significantly improve the corrosion resistance of the coating in a 3.5 wt% NaCl solution. Friction stir processing (FSP) was applied on the surface of Al–Fe as-sprayed layer to prepare Fe–Al intermetallic compounds on low carbon steel substrates [21]. By increasing the ESP passes, the grain boundaries in the microstructure can be decreased and the carbon content in the Widmänstatten ferrites can be increased, resulting in enhanced corrosion resistance in a 3.5 wt% NaCl solution. Fe–Al coating was prepared on low carbon steel substrates using mechanical alloying and subsequent heat treatment [22]. It is found that the lamellar structure in the coating can increase the corrosion resistance.

As far as we know, systematic investigation of the corrosion resistance of Fe–Al laser cladding coatings in a NaCl solution has not been reported. Since the corrosion properties of a coating is significantly affected by the fabrication method and the additive elements, it is necessary to study the corrosion behaviors of Fe–Al laser cladding coatings with different contents of Cr element. Herein, we fabricated Fe–Al–Cr coatings by a laser cladding process and systematically investigated their corrosion resistance in a 3.5 wt% NaCl solution. It was found that the addition of Cr atoms can effectively improve the corrosion resistance of the coatings in comparison with their Fe–Al counterpart. In particular, the Fe–Al–Cr coating with the addition of 5 at.% Cr exhibits the best corrosion resistance in a 3.5 wt% NaCl solution under both immersion and electrochemical conditions. Nevertheless, when the Cr additive is higher than 5 at.%, the Fe–Al–Cr coating will generate Al_8_Cr_5_ impurities in the boundaries, which is initially dissolved during corrosion tests. Thus, the corrosion resistance performance is decreased.

## 2 Experimental

### 2.1 Materials and methods

High-energy ball-milling technology was applied to synthesize the feedstock powder for laser cladding. The initial raw materials of iron, aluminum and chromium powders were all single metal powders with a purity of 99.9%. The metal powders exhibited good spherical structures with an average particle size of 2 μm. The specific preparation process was shown as follows: Firstly, four different proportions of Fe:Al:Cr powders were controlled as 72:28:0, 69:28:3, 67:28:5 and 65:28:7 (atomic ratio), respectively. Then, a high-energy Pulverisette 6 planetary mono-mill machine (Fritsch GmbH, Germany) was used to treat the powders to obtain solid solutions. Zirconium oxide balls were used to grind the powders. The specific ball milling process parameters were shown in Table .1. The obtained solid solution powders were denoted as Fe–28Al-xCr (x = 0, 3, 5, 7).

**Table 1:** Mechanical alloying process parameters for the Fe–Al–Cr composite powders.

Name	Parameter
Method	Dry milling
The ratio of stainless-steel ball to powder	10:1
Rotation rate	300 rpm
Ball milling time	50 h
Atmosphere	argon

Afterwards, the obtained Fe–Al-xCr powders were dispersed on 1045 steel substrates with dimensions of 150 mm × 150 mm × 5 mm, followed by putting into an ytterbium fiber laser system (YLR-200-SM) for laser cladding. The laser cladding parameters to fabricate the coatings were listed in Table 2. The obtained coatings were denoted as Fe–28Al-xCr (x = 0, 3, 5, 7) coatings, which were cut into smaller samples with dimensions of 15 mm × 15 mm × 5 mm.

**Table 2:** Process parameters for Fe–Al–Cr laser cladding coatings.

Name	Parameter
Spot size	70 μm
Laser power	180 W
Scanning speed	600 mm/s
Thickness of the pre-dispersed solid solution layer	50 μm

### 2.2 Characterization

A QUANT 200 scanning electron microscope equipped with an energy-dispersive spectroscope was used to characterize the morphology and elemental dispersion of the prepared solid solution powders and the laser cladding coatings. A Bruker D8 Advance X-ray diffractometer with Cu K_α_ radiation was applied to investigate the crystal structures of the solid solution powders and the laser cladding coatings before and after corrosion measurements.

A WS-2006 acoustic emission sensor with a Rockwell diamond indenter (radius: 0.2 mm) was performed for the scratch adhesion test. The load was increased from 0.05 N to 100 N with a fixed loading rate of 20 N/min. The speed of the indenter was 2 mm/min and the scratch length was 1 cm.

Immersion corrosion test and electrochemical corrosion test were used to investigate the corrosion resistance performances of the laser cladding coatings in a 3.5 wt% NaCl solution. For immersion corrosion test, the sizes and masses of the laser cladding coatings after ultrasonic cleaning and drying were measured. After immersing in a 3.5 wt% NaCl solution, the masses of the laser cladding coatings were measured every 5 days by a Satorius BS210S balance with a sensitivity up to 0.1 mg. The corrosion rate (*V*) of each coating was calculated according to equation (1), where S and t represent the surface area of coatings (m^2^) and the soaking time (h), while m_0_ is the initial weight (mg) and m_i_ is the mass of a coating after immersing for i days (mg).

(1) $$V=\frac{m_{0}-m_{i}}{St}$$

For electrochemical corrosion test, four Fe–Al–Cr coating samples were fused to copper wires to provide electrical connections, and the substrates as well as the edges were coated by resin to make sure that only the Fe–Al–Cr coating with an area of 10 mm × 10 mm was exposed to a 3.5 wt% NaCl aqueous solution at room temperature. The above obtained samples were used as the working electrodes, a Pt foil was used as a counter electrode, and a saturated calomel electrode was used as the reference electrode. Anodic polarization test and electrochemical impedance spectroscopy (EIS) test were carried out in a 3.5 wt% NaCl solution by a CHI7001 electrochemical workstation.

## 3 Results and discussion

### 3.1 Phase and morphology analysis of Fe–Al–Cr powders

**Fig. 1:**
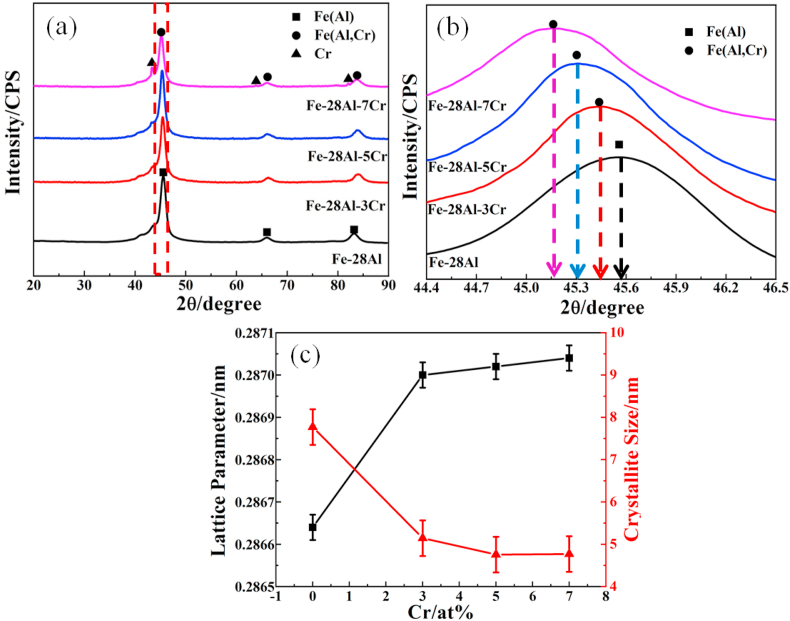
(a) XRD patterns of Fe–28Al-xCr powders, (b) enlarged figure of XRD patterns in the range of 44.4–46.5°, (c) the lattice parameters and average crystalline sizes of four kinds powders with different Cr addition.

Phase compositions and crystal structures of the Fe–28Al-xCr powders were characterized by X-ray diffraction (XRD). As shown in Fig. 1(a), three main peaks located at 45.57, 66.02, and 83.15° can be observed in the XRD pattern of Fe–28Al, which can be assigned to the Fe(Al) solid solution [23]. With the incorporation of Cr with an atomic ratio less than 5 at.%, no new peaks can be observed in the XRD patterns of Fe–28Al–3Cr and Fe–28Al–5Cr, indicating that Cr atoms also can be dispersed into the Fe lattice to form Fe(Al, Cr) solid solution. However, when the Cr content is 7 at.%, three new peaks assigned to Cr can be observed in the XRD pattern of Fe–28Al–7Cr, suggesting that the Cr content is over the solid solubility of α-Fe, and excessive Cr precipitate forms Cr impurity phase in Fe–28Al–7Cr.

Fig. 1(b) is the enlarged XRD patterns of the Fe–28Al, Fe–28Al–3Cr, Fe–28Al–5Cr, and Fe–28Al–7Cr samples in the range of 44.4–46.5° shown in Fig. 1(a). Compared to the Fe–28Al, the diffraction peaks of all Fe–Al-xCr powders exhibit a negative shift, indicating the increase of the d spacing due to the incorporation of Cr [24]. With the increase content of Cr, the peak shifts more negatively, suggesting that the d spacing is enlarged by the incorporation of more Cr atoms into the crystal lattice. The formation of Fe(Al,Cr) solid solution is attributed to the plastic deformation of powders to generate numerous vacancies during the high energy ball milling process, which accelerates the diffusion of Al and Cr atoms into the Fe lattice to form Fe(Al, Cr) disordered solid solution.

According to the Bragg equation (2dsinθ = nλ), the lattice spacing of the samples is increased from 0.28664 nm to 0.28704 nm with the incorporation of Cr from 0 to 7 at.% (Fig. 1(c)), which is due to the increase of the Cr atoms in the Fe lattice that enlarge the d spacing. In addition, the average crystalline sizes of the powders were calculated based on the Scherrer equation (D_hkl_ = Kγ/Bcosθ, where D_hkl_ refers to the average crystal size that is perpendicular to the (hkl) plane, K represents the Scherrer constant of 0.89, γ refers to 0.15406 nm which is the wavelength of X-ray, while B and θ represent the full width at half maximum (FWHM) and the diffraction degree of a XRD peak, respectively). As shown in Fig. 1(c), with the increase of the Cr content, the average crystalline size of the powder is reduced, indicating that the incorporation of Cr atoms can decrease the crystalline size of the Fe(Al, Cr) solid solution.

### 3.2 Morphology analysis of Fe–Al–Cr powders

**Fig. 2:**
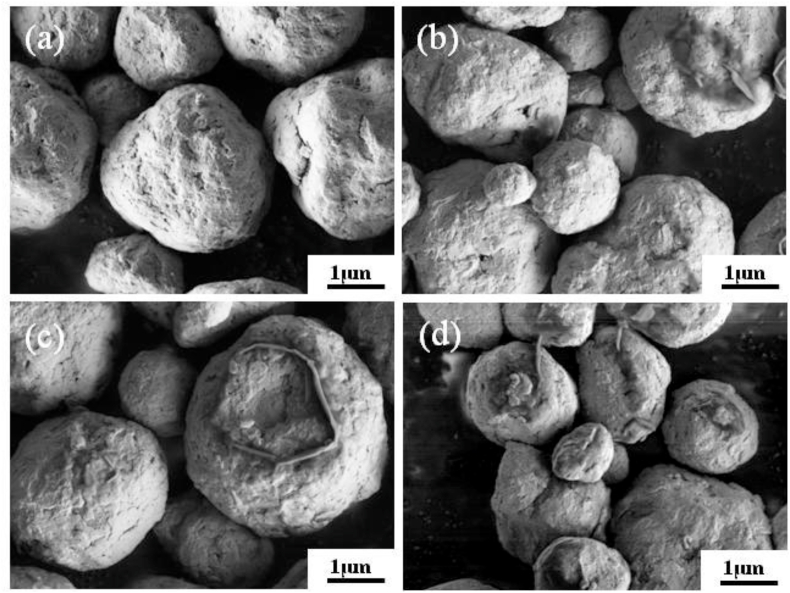
Morphologies of (a) Fe–28Al, (b) Fe–28Al–3Cr, (c) Fe–28Al–5Cr, and (d) Fe–28Al–7Cr powders.

Morphologies of the Fe–28Al-xCr samples were characterized by scanning electron microscopy (SEM). As shown in Fig. 2(a–d), the particles of all samples exhibit similar sphere-like structures with an average particle size of 1–2 μm, which is because all samples are obtained under the same high energy ball milling procedure. During the ball milling process, fracture, plastic deformation and cold welding occurred in the metal powders, which accelerate the diffusion of Al or Cr atoms through defects of the Fe particles, forming Fe(Al) and Fe(Al, Cr) solid solutions [25]. According to the elemental composition results listed in Table 3, the alloyed element ratios of all samples are close to the expected proportions.

**Table 3:** Elemental composition of Fe–28Al-xCr powders.

	Fe–28Al	Fe–28Al–3Cr	Fe–28Al–5Cr	Fe–28Al–7Cr
Fe/at.%	73.65	71.87	67.45	63.18
Al/at.%	26.35	25.18	27.52	28.63
Cr/at.%	0	2.95	5.03	8.19

### 3.3 Phase analysis of Fe–Al–Cr laser cladding coatings

**Fig. 3:**
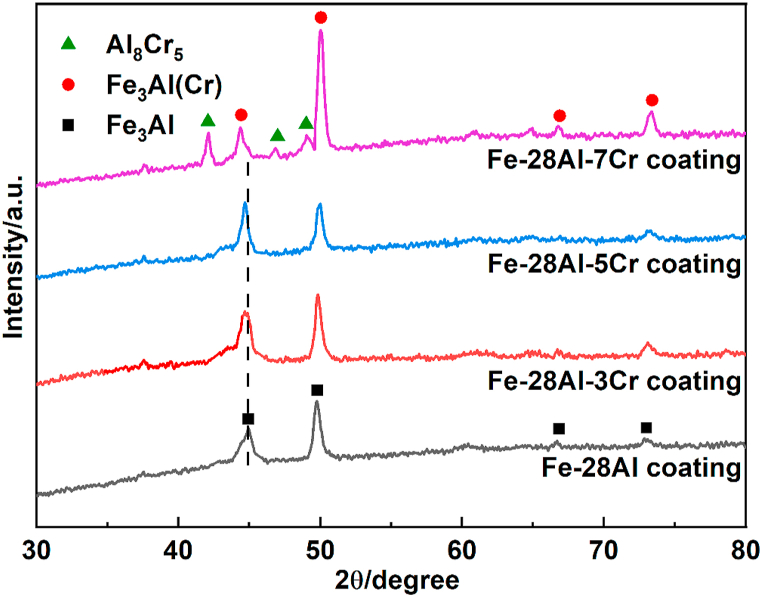
XRD patterns of the Fe–28Al, Fe–28Al–3Cr, Fe–28Al–5Cr, and Fe–28Al–7Cr laser cladding coatings.

The obtained solid solution powders were dispersed uniformly on 1045 steel substrates, and laser cladding was carried out to fabricate the corresponding coatings. Fig. 3 shows the XRD patterns of the Fe–28Al, Fe–28Al–3Cr, Fe–28Al–5Cr, and Fe–28Al–7Cr laser cladding coatings. All XRD peaks of Fe–28Al without any Cr addition can be assigned to the Fe_3_Al intermetallic compound [26]. When the Cr additive is lower than 5 at.%, all Cr atoms can be dispersed in the Fe_3_Al crystal lattice to form a Fe_3_Al(Cr) phase for the Fe–28Al–3Cr and Fe–28Al–5Cr coatings. It is worth noted that the Fe–28Al–7Cr coating is composed of Fe_3_Al(Cr) and Al_8_Cr_5_ [27]. Since the number of Cr atoms is higher than the maximum solid solubility of Cr in the Fe lattice (6 at.%) [28], the excessive Cr additive forms Al_8_Cr_5_ as the impurity phase in the coating.

### 3.4 Surface morphology analysis of Fe–Al–Cr laser cladding coatings

**Fig. 4:**
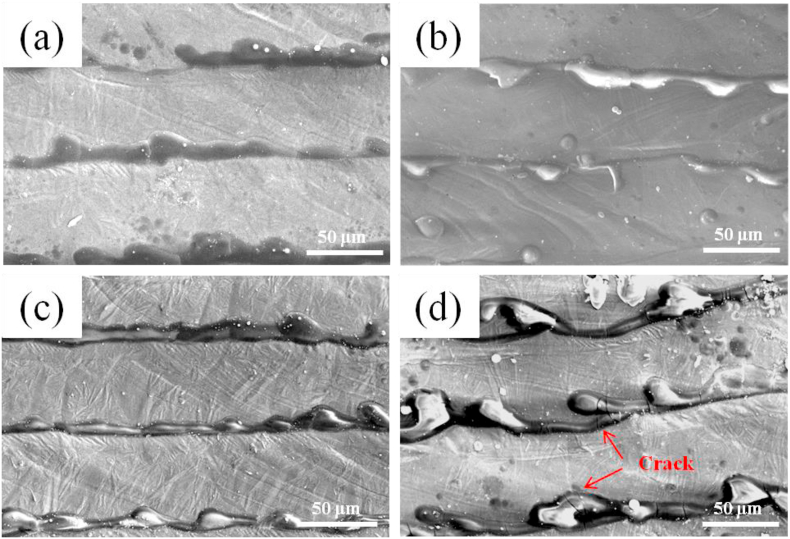
Surface morphologies of the (a) Fe–28Al, (b) Fe–28Al–3Cr, (c) Fe–28Al–5Cr, and (d) Fe–28Al–7Cr laser cladding coatings.

Surface morphologies of the Fe–28Al, Fe–28Al–3Cr, Fe–28Al–5Cr, and Fe–28Al–7Cr laser cladding coatings are shown in Fig. 4. Owing to the 30% lap rate, it is apparently that the scanning lines can be observed on the lap joints in all coatings. In particular, the surface morphologies of the Fe–28Al and Fe–28Al–3Cr coatings are similar, and some broken scanning lines can be observed (Fig. 4(a and b)). This phenomenon is attributed to the thermal stress in the coatings that originated from the rapid heating and quenching of the laser beam during the laser cladding process [29]. In contrast, the Fe–28Al–5Cr coating exhibits relatively uniform surface and no obvious defects can be observed in the surface and the scanning lines (Fig. 4(c)). However, some cracks can be observed in the Fe–28Al–7Cr coating (Fig. 4(d)). According to the XRD pattern (Fig. 3), the generation of Al_8_Cr_5_ may affect the cooling rate of the molten pool, and thus coarsening the microstructure after solidification. Thus, more thermal stress occurs in the Fe–28Al–7Cr coating, leading to the formation of cracks.

### 3.5 Cross-sectional microstructure analysis of Fe–Al–Cr laser cladding coatings

**Fig. 5:**
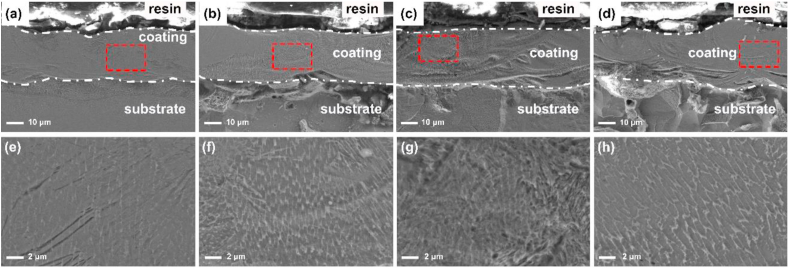
Cross-sectional microstructures of the Fe–28Al (a, e), Fe–28Al–3Cr (b, f), Fe–28Al–5Cr (c, g), and Fe–28Al–7Cr (d, h) laser cladding coatings. (e–h) are the enlarged images in the selective areas (red rectangles) in (a–d), respectively. (For interpretation of the references to colour in this figure legend, the reader is referred to the Web version of this article.)

Cross-sectional morphologies of the Fe–28Al, Fe–28Al–3Cr, Fe–28Al–5Cr, and Fe–28Al–7Cr laser cladding coatings are closely related to the temperature gradient of the liquid phase in the solid/liquid interface and the growth rate of the solid/liquid interface during solidification. In order to observe the microstructures of the coatings, the cross-sections of all coatings were etched by an ethanol solution containing 4% of nitric acid. As shown in Fig. 5(a–d), all coatings exhibit a similar thickness of approximately 30 μm, and the coatings are tightly deposited on the 1045 steel substrates. Fig. 5(e–h) are the partial enlargement of the selective areas in Fig. (a–d), respectively. It can be observed that all coatings exhibit similar columnar dendrites. With the incorporation of Cr atoms, the nucleation sites increase during solidification, leading to the formation of smaller grains in the microstructures. In addition, the incorporation of Cr atoms can decrease the melting point of the obtained solid solution powders, and thus affecting the temperature gradient and growth rate of the coatings during the solidification process. When the Cr additive is 5 at.%, the grain size is the smallest, which means the Fe–28Al–5Cr coating possesses great grain refinement strengthening effect [30]. Moreover, according to the XRD results (Fig. 3), no other impurity phases can be observed in the Fe–28Al–5Cr coating, suggesting that the incorporation of Cr has solid solution hardening effect on the coating.

### 3.6 Adhesion strength test of Fe–Al–Cr laser cladding coatings

**Fig. 6:**
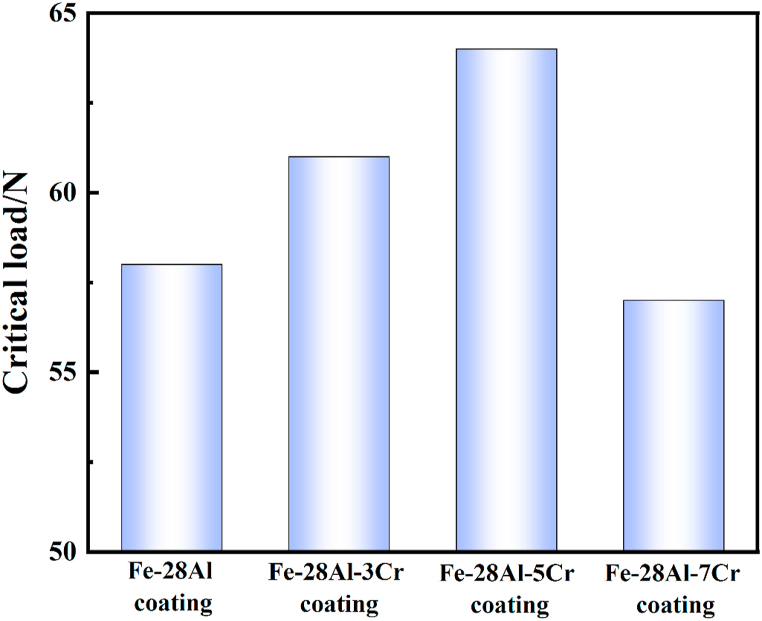
Scratch test results of the Fe–28Al, Fe–28Al–3Cr, Fe–28Al–5Cr, and Fe–28Al–7Cr laser cladding coatings.

Scratch test was performed to investigate the adhesion strength of the coatings. The load to obtain the first signal in the acoustic emission curve is defined as the critical load (L_c_), which is generally used to evaluate the adhesion strength of a coating on a substrate. The higher the L_c_ value, the stronger the adhesion strength. As shown in Fig. 6, L_c_ values of the Fe–28Al, Fe–28Al–3Cr, Fe–28Al–5Cr, and Fe–28Al–7Cr laser cladding coatings are 58, 61, 64 and 57 N, respectively, indicating that the Fe–28Al–5Cr coating has the highest adhesion strength with the 1045 substrate, which is attributed to the dense surface morphology without defects and the grain refinement strengthening effect.

### 3.7 Weight loss test

**Fig. 7:**
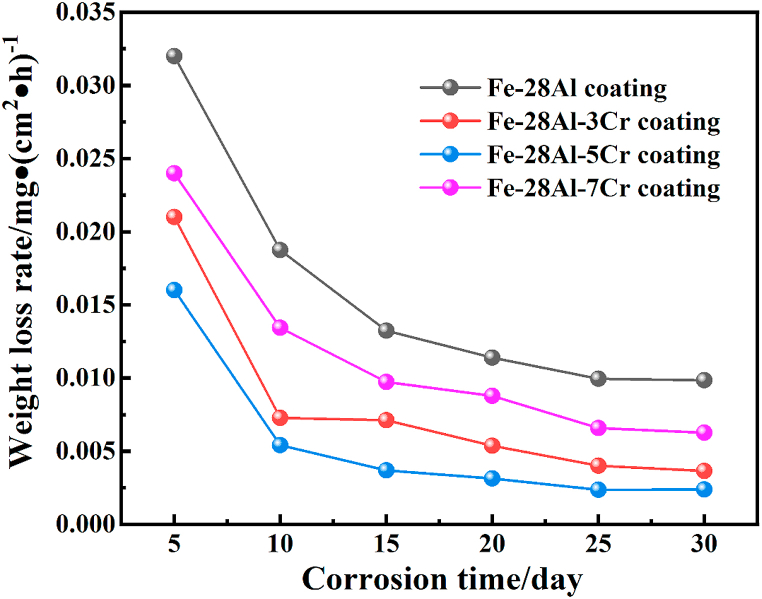
Weight loss curves of the Fe–28Al, Fe–28Al–3Cr, Fe–28Al–5Cr, and Fe–28Al–7Cr laser cladding coatings.

To investigate the corrosion resistance, the Fe–28Al, Fe–28Al–3Cr, Fe–28Al–5Cr, and Fe–28Al–7Cr laser cladding coatings were immersed in a 3.5 wt% NaCl solution for 30 days, and the weight loss rates of four samples were recorded every 5 days. As shown in Fig. 7, all coatings exhibit relatively large weight loss rates at the initial stage of the corrosion test. Owing to the attack of Cl^−^ ions, all coatings are difficult to form a stable passivation film at the beginning of the corrosion test, and the surfaces of the coatings suffer from repeated “passivation-damage-passivation” until reach equilibrium. After that, the weight loss rate is relatively stable during corrosion test.

Specifically, the Fe–28Al coating exhibits the largest weight loss rate of 0.032 mg m^−2^ h^−1^ in the initial 5 days, and obvious weight loss rates can be observed until the 25th day. With the incorporation of Cr, all Fe–28Al–Cr coatings show lower weight loss rates than their Fe–28Al counterpart during the immersion corrosion test, suggesting that the Cr additive can increase the corrosion resistance of the coating. In particular, the Fe–28Al–5Cr coating exhibits the lowest weight loss rate during the immersion corrosion test, with a value of only 0.016 mg m^−2^ h^−1^ in the initial 5 days. In addition, the weight loss rate becomes relatively stable at the 15th day, indicating that proper Cr additive can promote the formation of passivation film, thus exhibiting better corrosion resistance. However, excessive Cr additive lead to the decrease of corrosion resistance, possibly due to the formation of Al_8_Cr_5_ as the impurity phase in the Fe–28Al–7Cr coating.

**Fig. 8:**
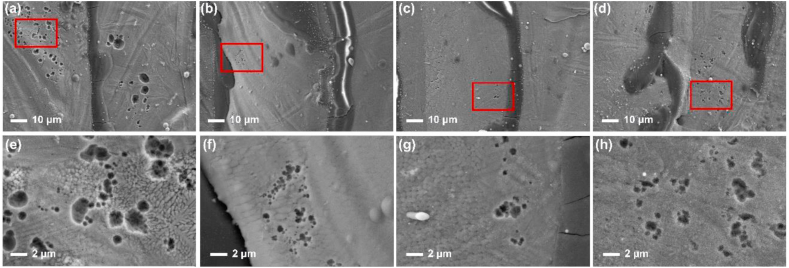
Corrosion morphology of the (a) Fe–28Al, (b) Fe–28Al–3Cr, (c) Fe–28Al–5Cr, and (d) Fe–28Al–7Cr laser cladding coatings after soaking in a 3.5 wt% NaCl solution for 30 days. (e–h) are the partial enlarged SEM images in the selective areas of (a–d).

Surface morphologies of the Fe–28Al, Fe–28Al–3Cr, Fe–28Al–5Cr, and Fe–28Al–7Cr laser cladding coatings after immersing in a 3.5 wt% NaCl solution for 30 days were observed by SEM. As shown in Fig. 8, some pits can be observed in all samples, exhibiting the typical pitting corrosion characteristics [31]. The Fe–28Al coating has the largest number of corrosion pits compared to other Fe–28Al–Cr coatings (Fig. 8(a–d)). In addition, large corrosion pits with a size of about 6.5 μm can be observed in the Fe–28Al coating (Fig. 8(a, e)). While for the Fe–28Al–3Cr coating, much less corrosion pits are observed, and the average size of the corrosion pits is around 0.58 μm (Fig. 8(b, f)). The Fe–28Al–5Cr coating exhibits the least corrosion pits (Fig. 8(c, g)), while more corrosion pits can be observed in the Fe–28Al–7Cr coating (Fig. 8(d, h)). Therefore, the Fe–28Al–5Cr coating exhibits the best corrosion resistance.

### 3.8 Electrochemical polarization test

**Fig. 9:**
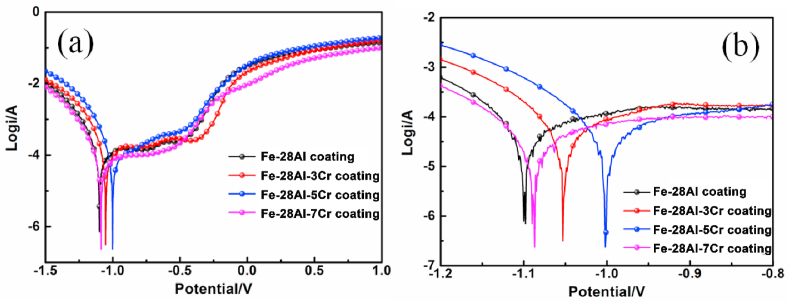
(a) Polarization curves of the Fe–28Al, Fe–28Al–3Cr, Fe–28Al–5Cr, and Fe–28Al–7Cr laser cladding coatings in a 3.5 wt% NaCl solution, and (b) the corresponding enlarged figure.

Fig. 9(a) illustrates the polarization curves of the Fe–28Al, Fe–28Al–3Cr, Fe–28Al–5Cr, and Fe–28Al–7Cr laser cladding coatings in a 3.5 wt% NaCl solution, and Fig. 9(b) is the enlargement of Fig. 9(a). The electrochemical parameters including the corrosion potentials (E_corr_), corrosion current densities (i_corr_), anodic Tafel slope (βa) and cathodic Tafel slope (βc) were extracted from these curves and listed in Table 4. It can be observed that the Cr additive significantly affects the polarization features of the coatings. All Fe–Al–Cr coatings exhibit more positive corrosion potentials while lower corrosion current densities than the Fe–28Al coating. The corrosion current density and the corrosion potential reflect the corrosion resistance of a coating. If the corrosion current density is lower and the corrosion potential is more positive, the coating has better corrosion resistance [32]. Hence, the polarization results demonstrate that the corrosion resistance of the Fe–28Al coating can be increased by the addition of Cr element. In particular, the Fe–28Al–5Cr coating shows the most positive corrosion potential and the lowest corrosion current density, suggesting the best corrosion resistance.

**Table 4:** Analysis results of potentiodynamic polarization curves.

Sample	E_corr_ (V)	i_corr_ (μA/cm^2^)	β_a_ (mV dec^−1^)	-β_c_ (mV dec^−1^)	R_p_ (kΩ/cm^2^)
Fe–28Al	−1.098	19.82	55	45	542.2
Fe–28Al–3Cr	−1.053	15.61	67	28	549.3
Fe–28Al–5Cr	−1.002	13.90	46	47	726.2
Fe–28Al–7Cr	−1.087	14.81	34	48	583.5

The values of polarization resistance (R_P_) of the coatings were calculated by equation (2)

(2) $$R_{p}=\frac{\beta_{a}\beta_{c}}{2.303i_{corr}\left(\beta_{a}+\beta_{c}\right)}$$

The results were listed in Table 4, which can be used to evaluate the surface resistance against the corrosion of aggressive ions (e.g. Cl^−^). According to the values of R_p_ for different coatings, the corrosion resistance of the Fe–28Al coating can be increased by the addition of Cr. The Fe–28Al–5Cr coating has the largest R_p_, which also proves the best corrosion resistance.

**Fig. 10:**
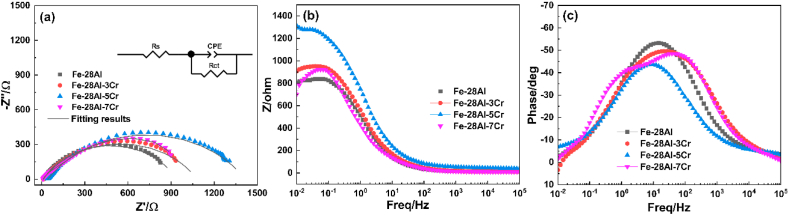
Electrochemical impedance spectroscopy plots of the Fe–28Al, Fe–28Al–3Cr, Fe–28Al–5Cr, and Fe–28Al–7Cr laser cladding coatings in a 3.5 wt% NaCl solution: (a) Nyquist plots, (b) Bode impedance modulus plots, and (c) Bode phase angle plots.

Electrochemical impedance spectroscopy (EIS) plots of the Fe–28Al, Fe–28Al–3Cr, Fe–28Al–5Cr, and Fe–28Al–7Cr laser cladding coatings were also collected in a 3.5 wt% NaCl solution. Generally, a larger arc radius in the Nyquist plot suggests a better corrosion resistance [33,34]. As shown in Fig. 10(a), all Fe–28Al–Cr coatings exhibit larger arc radius than their Fe–28Al counterpart, indicating that the Cr additive in the coating can effectively improve the corrosion resistance of the coating. In particular, the Fe–28Al–5Cr coating exhibits the largest arc radius amongst all coatings, suggesting the best corrosion resistance. All Nyquist plots of the coatings are composed of an arc, suggesting that all coatings exhibit passivation behaviors during the EIS measurement [35]. It can be observed that the impedance modulus of the Fe–28Al–5Cr coating is much larger than the other coatings at low frequency (Fig. 10(b)), which implies excellent barrier performance for the corrosion solution. The Bode phase angle plots of the Fe–28Al, Fe–28Al–3Cr, and Fe–28Al–5Cr coatings exhibit one phase angle maximum (Fig. 10(c)), suggesting the involvement of one time constant, which is relate to the electrolyte/coating interface. Interestingly, two phase angle maxima can be observed in the Bode phase angle plot of the Fe–28Al–7Cr coating, indicating the involvement of two time constants. Therefore, apart from the electrolyte/coating interface, there should be another interface in the Fe–28Al–7Cr coating during EIS test. According to the XRD results (Fig. 3), an impurity phase of Al_8_Cr_5_ is formed in the Fe–28Al–7Cr coating. Thus, the other interface should be due to the penetration of the electrolyte into the Fe–28Al–7Cr coating to create a Al_8_Cr_5_ impurity/electrolyte interface.

**Fig. 11:**
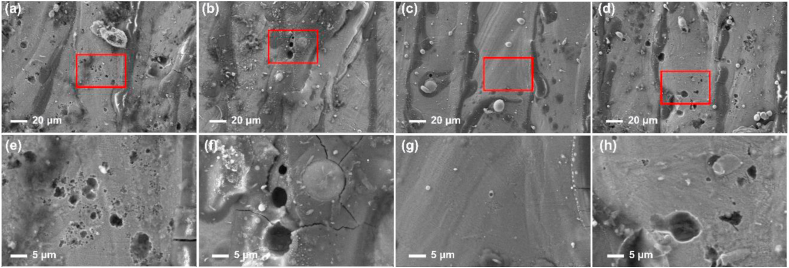
The surface morphology of the (a) Fe–28Al, (b) Fe–28Al–3Cr, (c) Fe–28Al–5Cr, and (d) Fe–28Al–7Cr laser cladding coatings after electrochemical test. (e–h) are the enlarged SEM images of the selective areas (red rectangle) in (a–d). (For interpretation of the references to colour in this figure legend, the reader is referred to the Web version of this article.)

Fig. 11 demonstrates the surface morphologies of the Fe–28Al, Fe–28Al–3Cr, Fe–28Al–5Cr, and Fe–28Al–7Cr laser cladding coatings after potentiodynamic polarization test. The Fe–28Al coating is severely corroded, and many corrosion pits can be observed (Fig. 11(a)). In particular, a large corrosion pit with a size of about 6 μm is observed (Fig. 11(e). The Fe–28Al–3Cr laser cladding coating exhibits much less corrosion pits (Fig. 11(b)). However, the size of the corrosion pits is around 8 μm and some cracks can be observed (Fig. 11(f)). In contrast, the Fe–28Al–5Cr coating exhibits a relatively good surface (Fig. 11(c)), and no obvious corrosion pits can be observed (Fig. 11(g)), indicating the excellent corrosion resistance. While for the Fe–28Al–7Cr coating, some obvious corrosion pits can be observed (Fig. 11(d, h)). Therefore, the Fe–28Al–5Cr coating has the best corrosion resistance.

**Fig. 12:**
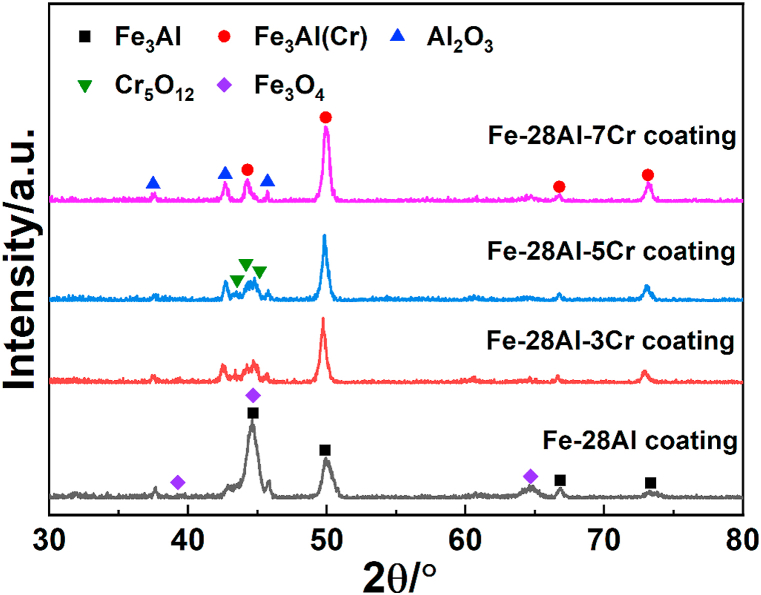
XRD patterns of the Fe–28Al, Fe–28Al–3Cr, Fe–28Al–5Cr, and Fe–28Al–7Cr laser cladding coatings after electrochemical corrosion.

To further understand the underlying mechanism for the corrosion resistance, XRD was conducted to characterize the corrosion products on the surfaces of the Fe–28Al, Fe–28Al–3Cr, Fe–28Al–5Cr, and Fe–28Al–7Cr laser cladding coatings after potentiodynamic polarization test. As shown in Fig. 12, Al_2_O_3_ signals can be observed in the XRD pattern of the Fe–28Al coating. In addition, the relative intensity of the peak at around 44.6° of the Fe–28Al coating after potentiodynamic polarization test is much higher compared to the as-prepared coating (Fig. 3), which is attributed to the formation of Fe_3_O_4_ that has a characteristic peak at the similar position (44.76°). While for the Fe–28Al–3Cr and Fe–28Al–5Cr coatings, a new phase of Cr_5_O_12_ can be observed, which can effectively hinder corrosion reaction in the 3.5 wt% NaCl solution. However, the characteristic peaks of Cr_5_O_12_ cannot be apparently observed in the XRD pattern of the Fe–28Al–7Cr coating, possibly due to the Al_8_Cr_5_ impurity that acts as a sacrificial micro-anode. Therefore, proper Cr additive can generate both Al_2_O_3_ and Cr_5_O_12_ as the compact passive films on the surface of the Fe–Al–Cr coating in the 3.5 wt% NaCl solution, which can significantly enhance the corrosion resistance.

## 4 Conclusions

In summary, Fe–28Al-xCr coatings with different Cr contents (0, 3, 5, and 7 at.%) were prepared by a laser cladding process and the effect of Cr additive on the corrosion resistance in a 3.5 wt% NaCl solution was systematically studied. It was found that the Cr additive has solid solution hardening and grain refinement strengthening effects on the coatings, which can effectively increase the corrosion resistance. In particular, the Fe–28Al–5Cr coating can incorporate all Cr atoms into the Fe matrix without phase segregation, which forms a coating with excellent quality and interfacial adhesion. Both immersion and electrochemical corrosion tests demonstrated that the Fe–28Al–5Cr coating exhibits the best corrosion resistance in a 3.5 wt% NaCl solution due to the formation of a passivation film composed of Al_2_O_3_ and Cr_5_O_12_. Nevertheless, when the Cr additive is increased to 7 at.%, an impurity phase of Al_8_Cr_5_ can be observed, which will be initially corroded during corrosion measurements, resulting in reduced corrosion resistance.

## Author contribution statement

Xixi Luo: Conceived and designed the experiments; Performed the experiments; Wrote the paper.

Jing Cao: Analyzed and interpreted the data.

Fangli Yu, Yalong Zhang, Xin Li: Contributed reagents, materials, analysis tools or data.

Jianjiang Tang: Analyzed and interpreted the data; Wrote the paper.

Hui Xie: Conceived and designed the experiments; Wrote the paper.

## Funding statement

This work was supported by the National Natural Science Foundation of China [52005384, 51871173], the fund of the State Key Laboratory of Advanced Processing and Recycling of Non-ferrous Metals, Lanzhou University of Technology [SKLAB02019003], the Innovative Talents Promotion Project of Shaanxi Province [2022KJXX-45], the Natural Science Basic Research Program of Shaanxi [2020JQ-909, 2020JM-631, 2020JQ-906], the Scientific Research Program Funded by the Shaanxi Provincial Education Department [20JK0693], the Youth Innovation Team of Shaanxi Universities, Open Fund project of Shaanxi Provincial Key Laboratory of Hydraulic Technology [YYJS2022KF07].

## Data availability statement

Data will be made available on request.

## Declaration of interest’s statement

The authors declare no competing interests.
